# Using a Large Language Model to Identify Adolescent Patient Portal Account Access by Guardians

**DOI:** 10.1001/jamanetworkopen.2024.18454

**Published:** 2024-06-25

**Authors:** April S. Liang, Shivam Vedak, Alex Dussaq, Dong-Han Yao, Keith Morse, Wui Ip, Natalie M. Pageler

**Affiliations:** 1 Division of Clinical Informatics, Stanford University School of Medicine, Palo Alto, California; 2 Department of Pediatrics, Stanford University School of Medicine, Palo Alto, California

## Abstract

This diagnostic/prognostic study assesses the ability of a large language model (LLM) to detect guardian authorship of messages originating from adolescent patient portals.

## Introduction

The 21st Century Cures Act mandates electronic health record (EHR) access for patients and their legal representatives. In its balance, the Health Insurance Portability and Accountability Act (HIPAA) and state minor consent laws stipulate that adolescents can consent to specific health services and have certain privacy rights over related data.^1,2^ To reconcile these legal requirements, patient portals offer differential access to the health record for adolescent vs parent and/or guardian proxy accounts. However, 64% to 76% of adolescent accounts are directly accessed by guardians,^3^ jeopardizing confidentiality and potentially affecting adolescents’ willingness to engage with care.^4^ Our institution developed a rules-based natural language processing (NLP) algorithm to detect direct guardian access of adolescents’ primary accounts through message content analysis^3^; however, low sensitivity and manual workflow limited its utility. Large language models (LLMs) have excelled in natural language-based medical tasks,^5^ and emerging EHR–LLM integrations provide opportunities for seamless workflow. In this study, a LLM’s ability to detect guardian authorship of messages originating from adolescent patient portals was tested.

## Methods

This single-site diagnostic/prognostic study describes the GPT-4 (Open AI; model gpt-4-32k-0613) LLM’s performance at identifying parent- and/or guardian-authored portal messages. Messages from adolescent patient portal accounts at Stanford Children’s Health between June 1, 2014, and February 28, 2020, were sampled and manually reviewed for authorship as described in the study by Ip et al.^3^ Two prompts were iteratively engineered on a stratified random subset of 20 messages until perfect performance (100% sensitivity and specificity) was achieved: one focused on authorship identification (single task, eMethods in Supplement 1) and another that generated a response to the message and identified authorship (multitask, eMethods in Supplement 1). Both prompts were tested on remaining messages using our institution’s personal health information–compliant LLM (eFigure in Supplement 1) with our NLP algorithm's performance as a benchmark (eMethods and eTable in Supplement 1). To account for correlated data, performance on 1 randomly selected message per patient was analyzed (eMethods in Supplement 1). Positive predictive values (PPV) and negative predictive values (NPV) were calculated from the tested sample, then mathematically modeled on varying prevalences (eMethods in Supplement 1). The 95% CIs were calculated using the Clopper-Pearson exact method. Statistical analysis was performed with JavaScript ECMAScript 2023 from December 2023 to April 2024.

## Results

Of the 2088 test messages, 1500 (71.8%) were labeled as parent- or guardian-authored and 588 (28.2%) as patient-authored. The single-task LLM achieved a sensitivity of 98.1% (95% CI, 97.3%-98.8%), and the multitask LLM achieved a sensitivity of 98.3% (95% CI, 97.5%-98.9%). The single-task LLM achieved a specificity of 88.4% (95% CI, 85.6%-90.9%); and the multitask LLM achieved a specificity of 88.9% (95% CI, 86.1%-91.4%) (Table). This corresponded to PPV and NPV greater than 95% for multitask LLM, and the classifiers’ PPV and NPV exceeded 90% on the previously reported prevalence range^3^ (Figure). Single-task and multitask classifiers performed statistically identically, and removing correlated data did not significantly affect classifier performance (Table).

**Table.** Performance Characteristics of the LLM Classifiers^a^

Test characteristic	% (95% CI)			
	Single-task LLM	Multitask LLM	Single-task LLM single message	Multitask LLM single message
Sensitivity	98.1 (97.3-98.8)	98.3 (97.5-98.9)	98.3 (94.1-99.8)	98.3 (94.1-99.8)
Specificity	88.4 (85.6-90.9)	88.9 (86.1-91.4)	84.2 (74.0-91.6)	84.2 (74.0-91.6)
PPV	95.6 (94.4-96.6)	95.8 (94.7-96.7)	90.8 (84.4-95.1)	90.8 (84.4-95.1)
NPV	94.9 (92.7-96.6)	95.4 (93.3-97.0)	97.0 (89.5-99.6)	97.0 (89.5-99.6)

Abbreviations: LLM, large language model; NPV, negative predictive value; PPV, positive predictive value.

^a^ Performance was measured on the full test set of messages (2088 messages) and on a single random message per patient account (197 messages) in order to remove effects of correlated data.

**Figure.**
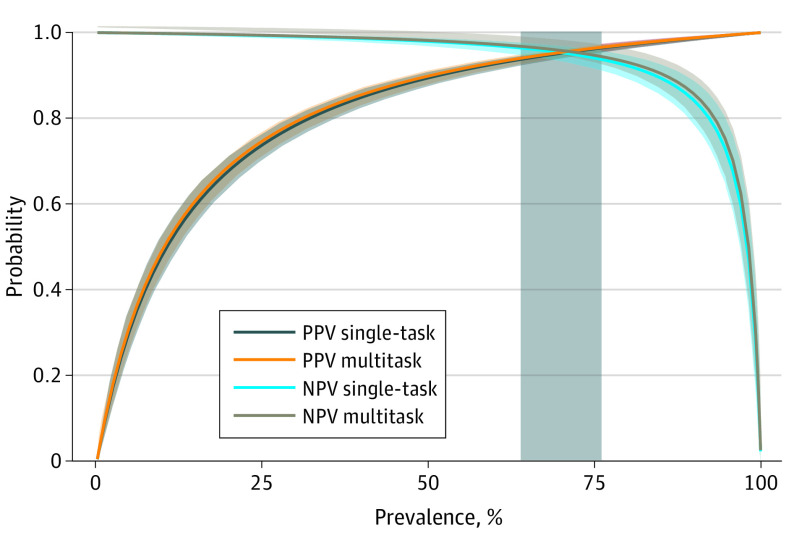
Positive Predictive Value (PPV) and Negative Predictive Value (NPV) Performance With 95% CI of the Large Language Model Classifiers Across Varying Prevalence of Parent-Authored Messages Prevalence in the randomly sampled dataset was 71.8%; prior studies have estimated that prevalence ranges from 64%-76% (as shown in the shaded horizontal).

## Discussion

This study’s LLM-based classifiers accurately detected guardian authorship of messages sent from an adolescent patient portal, achieving PPV and NPV exceeding 95%. This LLM had significantly better sensitivity and NPV than our current NLP algorithm and could enhance adolescent confidentiality, identifying more instances of direct guardian access with a relatively small increase in false positives. Our head-to-head comparison of different prompts reassuringly showed no performance deterioration despite the added cognitive burden of drafting a response in the multi-task large language model classifier. Therefore, these results suggest that EHR integrations can perform both tasks in a single LLM interaction, presenting a scalable application for clinical use. Limitations included single-site data, exclusions of non-English messages, and small number of unique patients. Additionally, expert review may have misidentified the author. Challenges for implementation included the need for an HIPAA-compliant LLM instance, accounting for instances where patients permitted direct portal access by parents and/or guardians, and thoughtful communication around false-positive cases. Ultimately, reliable identification of nonpatient-authored messages has implications beyond adolescent medicine. Among adults, care partners commonly access patient portals using the patient’s credentials,^6^ especially relevant for geriatric patients or individuals with developmental differences. Our results found that this study’s LLM has potential in improving safeguards for patient confidentiality.

## Notes

Supplement 1. **eMethods.** Rules-Based NLP Algorithm **eTable.** Performance Characteristics of Existing NLP Classifier **eFigure.** Study Design

Supplement 2.
Data Sharing Statement
