# Supplementary material for: Using a Large Language Model to Identify Adolescent Patient Portal Account Access by Guardians
Source: JAMA Netw Open. 2024 Jun 25;7(6):e2418454. doi: 10.1001/jamanetworkopen.2024.18454 (PMC11200138; doi:10.1001/jamanetworkopen.2024.18454)
Supplement: Supplement 2. — Data Sharing Statement [file jamanetwopen-e2418454-s002.pdf]

Data Sharing Statement

Liang. Using a Large Language Model to Identify Adolescent Patient Portal Account Access by Guardians. *JAMA Netw Open*. Published June 25, 2024.
doi:10.1001/jamanetworkopen.2024.18454

Data
Data available: No

Additional Information
Explanation for why data not available: Dataset includes patient-delivered messages containing PHI.
